# The impact of the expression signatures of LncRNAs *HBBP1* and *XIST* on the diagnostic significance of patients with β-Thalassemia

**DOI:** 10.1007/s00277-026-06751-5

**Published:** 2026-02-04

**Authors:** Abdallah M. Gameel, Shimaa Abdelsattar, Zeinab A. Kasemy, Mai El-Sayad Abd El-Hamid, Eman Masoud Abd ElGayed, Basma M. Abdelgawed, Amira Fathy El-Fiky, Mariam E. Labib, Sabry M. Abdelmageed, Mahmoud Ahmed El Hawy, Yasmin Mohsen, Hanan M. Bedair

**Affiliations:** 1 Clinical Pathology Department, National Cancer Institute, Cairo University, Kasr Al-Aini Street, Fom El Khalig Square, Cairo, 11796 Egypt; 2 Clinical Biochemistry and Molecular Diagnostics Department, National Liver Institute, Menoufia University, Menoufia, Egypt; 3 Department of Public Health and Community Medicine, Faculty of Medicine, Menoufia University, Shebine Elkoum, Egypt; 4 Pediatrics Department, Faculty of medicine, Menoufia University, Menoufi, Egypt; 5 Medical Biochemistry and Molecular Biology Department, Faculty of Medicine, Menoufia University, Menoufia, Egypt; 6 Faculty of medicine, Menoufia National University, Menoufia, Egypt; 7 Medical Biochemistry and Molecular Biology Department, Faculty of Medicine, Ain Shams University, Cairo, Egypt; 8 Clinical Pathology Department, National Liver Institute, Menoufia University, Menoufia, Egypt

**Keywords:** Β-thalassemia, LncRNA-HBBP1, LncRNA-XIST, RT-qPCR

## Abstract

β-thalassemia is an inherited blood disorder with long-term associated complications. The purpose of this study was to evaluate the clinical significance of the *lncRNA-HBBP1* and *lncRNA-XIST* expression profiles in the diagnosis of β-thalassemia patients. One hundred children patients with β-thalassemia participated in this case-control study: 50 patients diagnosed as Beta Thalassemia Major (β-TM) and 50 patients diagnosed as Beta Thalassemia Intermedia (β-TI) groups. Furthermore, there were 50 children as healthy control group. Assessment of both genes’ expression was performed by RT-qPCR. The findings displayed that both *lncRNA-HBBP1 and lncRNA-XIST* were highly expressed within the β-TM group than in the β-TI and the control groups (*P* < 0.001 for both). The *lncRNA-HBBP1* and *lncRNA-XIST* expression were significantly higher in β-thalassemia patients presented with jaundice, thalassemia facies, or organomegaly (*p* < 0.001 for all). In addition, *lncRNA-XIST* expression was significantly higher in β-thalassemia patients with splenectomy (*p* = 0.002). Spearman correlation revealed that the expression of both genes was significantly correlated with HbF % in β-TM and β-TI groups (*p* < 0.001 for both). Based on the ROC curve analysis, the sensitivity of *lncRNA-HBBP1* and *lncRNA-XIST* for discriminating the β-TM group from the β-TI group was 82% and 80%, respectively. Collectively, the examined lncRNAs could offer novel biomarkers for β-thalassemia disorder once confirmed in extensive upcoming investigations.

## Introduction

Thalassemia is a progressive, heterogeneous set of disorders affecting hemoglobin (Hb) synthesis, caused by single-gene defects. It is anticipated that in 20 years, 900,000 newborns will have clinically significant thalassemia disorders [1]. Thalassemia is either alpha (α) or beta (β) thalassemia. Both types are caused by gene mutations in the α or β globin protein, resulting in reduced Hb synthesis and induced hypoxia-related symptoms [2]. Different types of β-thalassemia are categorized by the mutation location in the human globin gene. Mutations that totally inactivate the β gene prevent β-globin synthesis. β-thalassemia is classified into: (1) β-thalassemia major (β-TM), commonly known as Cooley’s anemia, is a severe form of anemia requiring regular blood transfusions, triggered by a mutation of the β-globin gene (either homozygous or compound heterozygous). It is associated with various clinical manifestations such as distinct facial features, jaundice, hepatosplenomegaly, and growth interruption. The symptoms occur between 6 and 24 months. (2) β-thalassemia intermedia (β-TI), also known as Non-Transfusion-Dependent Thalassemia (NTDT), includes patients require occasional transfusions for short periods and present later than β-TM. (3) β-thalassemia minor, also known as asymptomatic carrier state, which results from a mutation in one copy of the β-globin gene and usually manifests as mild anemia without symptoms. β-TI falls between β-thalassemia minor and β-TM in terms of clinical severity [2].

Many criteria contribute to the diagnosis of thalassemia, including physical examination, complete blood count (CBC), and Hb electrophoresis. However, there is a requirement to enhance thalassemia detection and diagnosis through a combination of laboratory tests [3, 4]. It is still unclear how different β-thalassemia phenotypic presentations can result from the same beta-globin gene alterations. However, epigenetic regulators such as long non-coding RNAs (lncRNAs) can affect Hb synthesis, which has been clarified in many studies [5–8]. Growing evidence indicates that this category of non-coding RNAs, consisting of more than 200 nucleotides, has a role in a wide range of biological processes in normal and pathological conditions. These activities include various cellular developmental processes, X-chromosome inactivation, cell differentiation, genomic imprinting, and immune response [9]. During erythropoiesis, many tissue-specific lncRNAs are expressed [9–11] and are involved in hematopoiesis and the etiology of blood disorders [12, 13]. In normal cells, lncRNA prevents miRNA binding to maintain hemoglobin F (HbF) levels, but in beta-thalassemia, dysregulated lncRNAs raise HbF. High HbF levels may be caused by lncRNA activating HB Subunit Epsilon 1 (HbE1) and haemopoietic cell lineage-inducible molecule [14].

X-inactive specific transcript (*XIST*) is a non-coding RNA within the X chromosome and is the most vital regulator of X-chromosome inactivation in mammals [15]. It is essential for cancer cell proliferation and differentiation [16]. Specifically, it may play an oncogenic role in hematological diseases due to gene dysregulation caused by heterochromatin instability [17]. Additionally, it has been demonstrated that *lncRNA-XIST* expression is linked to the development of some tumors that are not related to sex. However, the diagnostic significance of *lncRNA-XIST* in thalassemia has not been elucidated [18].

Hemoglobin subunit β pseudogene 1 (*HBBP1*) is situated on chromosome 11 adjacent to lncRNA- Beta Globin Locus Transcript 3 (*BGLT3*) and adult δ-globin (hemoglobin delta (HbD) [18]. Although *lncRNA-HBBP1* did not encode a protein, it showed elevated expression in erythroblasts and increased dramatically throughout erythroid differentiation. Despite not being translated, the *lncRNA-HBBP1* transcript can attach to an RNA-binding protein, heterogeneous nuclear ribonucleoprotein A1 (*HNRNPA1*), which can intercede the RNA decay of T-cell acute leukemia protein 1 (*TAL1*), an essential erythropoiesis regulator. Therefore, *lncRNA-HBBP1* stabilizes *TAL1* mRNA and binds *HNRNPA1* competitively to induce erythropoiesis [19]. However, the diagnostic significance of *lncRNA-HBBP1* in thalassemia patients requires further investigations [20].

This study aimed to estimate the clinical significance of *lncRNA-HBBP1* and *lncRNA-XIST* expression profiles in the diagnosis of β-thalassemia patients, which could aid medical professionals and researchers in better comprehending the pathophysiology of this disorder.

## Materials and methods

### Study design

This case-control research comprised 100 children with β-thalassemia and 50 healthy controls. Patients with β-thalassemia were divided into 50 with β-TM (Transfusion-Dependent Thalassemia (TDT)) and 50 with β-TI (Non-Transfusion-Dependent Thalassemia (NTDT)). The diagnosis of β-thalassemia was confirmed using CBC, Hb electrophoresis, and HbF % quantification. The β-thalassemia were categorized based on clinical severity and blood transfusion requirements. Patients represented with hemoglobinopathies or other kinds of anemia in clinical or laboratory settings and acute infections during blood collection were excluded. The controls were healthy, age- and sex-matched children. Their siblings joined them at the pediatric outpatient clinic. Their exclusion criteria were no family history of hemoglobinopathies or blood transfusions. Study participants were recruited from the Hematology Pediatric Unit, Menoufia University Hospital, Egypt, between November 2021 and September 2023. The Menoufia University Faculty of Medicine Ethical Scientific Committee accepted this study (IRB approval number and date: 11/2022 PEDI 8). The study followed the Helsinki Declaration for human studies. All research participants or guardians gave informed written consent. All patients and controls underwent a clinical examination. CBC, Hb electrophoresis, and serum ferritin assays were performed.

### Clinical and child growth evaluation of the patients

A standardized interview form was used to collect data from all research participants’ medical records during visits to pediatric clinics or hospitalization for routine blood transfusions. We used a child’s height for age to identify cases of stunted or excessive growth. A child’s weight for age was used to evaluate if they were as underweight or severely underweight. Weight-for-height was utilized to detect wasting and obese children. A child’s BMI was used to measure overweight and obesity. A World Health Organization (WHO) algorithm was used to calculate Egyptian children’s z-scores using growth charts [21].

### Samples collection

Blood sample collection was performed on all participants in different groups; under complete septic conditions, 6 ml of venous blood was drawn. Four ml was brought into an ethylene diamine tetra acetic acid (EDTA) vacutainer; 2 ml was used for assessment of CBC and Hb electrophoresis, and the remaining 2 mL were centrifuged and separated then stored at −80◦C until the time of assessment *lncRNA-HBBP1* and *XIST* expression in plasma. The other 2 ml was placed in plain test tubes for serum ferritin assay.

### Assessment of *lncRNA-HBBP1* and *lncRNA-XIST* gene expression by RT-qPCR

Total RNA, including lncRNA, was extracted from the plasma samples according to the manufacturer’s guidelines of miRNeasy Mini Kit (Qiagen GMbH, Hilden, Germany, cat. No. 217004). The purity and concentration of the extracted RNAs were assessed using a NanoDrop 2000 spectrophotometer from Thermo Scientific (Waltham, MA (Massachusetts), USA). The extracted RNAs were kept at − 80 ◦C until it was required. One µg of the extracted RNAs underwent complementary DNA (cDNA) reverse transcription using a SensiFASTTM cDNA Synthesis Kit (Bioline, USA), depending on the manufacturer’s guidelines. Real-time quantitative reverse transcription polymerase chain reaction (RT-qPCR) was employed to assess the expressions of *lncRNA-HBBP1* and *lncRNA-XIST*, with glyceraldehyde 3-phosphate dehydrogenase (*GAPDH*) as a reference gene, utilizing the SensiFAST™ SYBR Lo-ROX Kit (Bioline, USA). Relative quantification (RQ) of *lncRNA-HBBP1* and *lncRNA-XIST* gene expression was conducted utilizing specifically designed primers (Midland, TX, USA), which were authorized by the National Centre for Biotechnology Information (NCBI). The *lncRNA-HBBP1* primers were: Forward, 5′-TCACGGATGACCTCAAAGGCAC-3′ and Reverse, 5′- AATCCTCGCTGAAGTGGGTTGC-3′. The *lncRNA-XIST* primers were: Forward, 5′-GGCTTAGGGCTAGTCGTTTGT-3′ and Reverse, 5′-TTCCTCTGCCTGACCTGCTAT − 3′. The GAPDH primers were: Forward, 5′-GAAGGTGAAGGTCGGAGTC-3′ and Reverse, 5′-GAAGATGGTGATGGGATTTC-3′. The reaction volume was 20 µl, comprising 6 µl of template cDNA, 10 µl of SYBR Green Master Mix, 1.5 µl of forward primer, 1.5 µl of reverse primer, and one µl of nuclease-free water. The *lncRNA-HBBP1*, *lncRNA-XIST*, and *GAPDH* were amplified utilizing the ABI7500 real-time PCR apparatus (Applied Biosystems, USA) software version 2.0.1 under the specified cycling conditions: an initial denaturation at 95 °C for 5 min, followed by 45 cycles consisting of 95 °C for 20 s, 60 °C for 30 s, 72 °C for 1 min, concluding with a final extension at 72 °C for 10 min. RQ of both *lncRNA-HBBP1* and *lncRNA-XIST* genes was achieved by normalizing their amount to the GAPDH gene and comparing them to the control using the 2^−∆∆CT^ method [22].

### Sample size

A statistical power analysis was conducted based on estimation of sample size derived from the present study (*N* = 150), comparing Group 1 with Group 3. The effect size (ES) for *lncRNA-HBBP1* in the study was 13.60, which is considered very large, according to Cohen’s (1988) criteria, with an alpha of 0.05 and sample size of 50 in each group. A post-hoc power analysis conducted utilizing this effect size (GPower 3.1), resulting in an approximate (1-_β_) = 1.0. A sample size of 50 in each group is sufficient to achieve for the main goal of this study.

### Statistical analysis

Data were analyzed with IBM SPSS Statistics version 20 (SPSS Inc., Chicago, IL). The Chi-square test was applied to analyze the relationship between qualitative variables, while Fisher’s exact test was utilized when more than 20% of cases have frequencies below 5. Comparisons of quantitative data between two groups were conducted using either the Student’s t-test or the Mann-Whitney test (non-parametric), as appropriate. Comparisons among three groups were done using either ANOVA or the Kruskal-Wallis test (non-parametric), as appropriate. The Spearman-rho was used to assess the association between NR_001589.1 (*lncRNA-HBBP1)* or *lncRNA-XIST* and other numerical variables. The Receiver Operator Characteristic Curve (ROC) displays the association between sensitivity and specificity at various cut-off thresholds for NR_001589.1 (*lncRNA-HBBP1)* or *lncRNA-XIST* in the context of thalassemia major vs. thalassemia intermediate. A p-value ≤ 0.05 was considered significant.

## Results

###  The clinical and laboratory features

A total of 150 participants took part in this study, classified into three groups: 50 patients with β-TM, 50 patients with β-TI, and the remaining 50 healthy children were recruited as controls. The general features among the studied groups are illustrated in (Table 1). The weight and BMI significantly decreased among both β-TM and β-TI in comparison to the control group (*p* = 0.005, 0.05 and *P* = 0.01, 0.001, respectively). Also, BMI was notably decreased among the β-TM group compared to the β-TI group (*p* = 0.006). Regarding clinical features of the patients in the study, ‘Jaundice, frequency of blood transfusion, organomegaly, splenectomy, and thalassemia facies’ were significantly higher among β-TM patients than in β-TI patients (P < 0.001 for all) as illustrated in (Table 2).

**Table 1:** The general features among the studied groups

Features	β-TM group (No.=50)	β-TI group (No.=50)	Control group (No.=50)	*P* value	Post hoc test
Age (years) Median (IQR)	8 (4.4–12)	9 (5–11.4)	8.7 (4.7–11)	0.83	P1 = 0.58
					P2 = 0.84
					P3 = 0.64
Sex				0.85	P1 = 0.68
Male	28 (56.0%)	30 (60.0%)	29 (58.0%)		P2 = 0.84
Female	22 (44.0%)	20 (40.0%)	21 (42.0%)		P3 = 0.83
Height (Cm) Mean ± SD	116.8 ± 19.5	121.3 ± 18.5	123.5 ± 25.6	0.29	P1 = 0.29
					P2 = 0.12
					P3 = 0.62
Weight (Kg) Median (IQR)	21 (15–30)	22 (18–30)	25 (18–46)	**0.01**	P1 = 0.24
					**P2 = 0.005**
					**P3 = 0.05**
BMI Mean ± SD	15.2 ± 0.55	15.9 ± 0.77	17.9 ± 1.9	**< 0.001**	**P1 = 0.006**
					**P2 < 0.001**
					**P3 < 0.001**

β-TM: Beta thalassemia major, β-TI: Beta thalassemia intermedia, IQR: Interquartile range, SD: Standard deviation, BMI: Body mass index

P1: β-TM group versus β-TI group

P2: β-TM group versus control group

P3: β-TI group versus control group

**Table 2:** Comparison of clinical characteristics of β- thalassemia patient groups

Clinical characteristics	β-TM group (No.=50)	β-TI group (No.=50)	*P* value
Pallor			
Yes	49 (98.0%)	50 (100%)	1.00
No	1 (2.0%)	0 (0.0%)	
Jaundice			
Yes	41 (82.0%)	8 (16.0%)	**< 0.001**
No	9 (18.0%)	42 (84.0%)	
Thalassemia facies			
Yes	30 (60.0%)	0 (0.0%)	**< 0.001**
No	20 (40.0%)	50 (100%)	
Frequency of blood transfusion/year Mean ± SD	9.6 ± 3.1	2.5 ± 0.7	**< 0.001**
Organomegaly			
Yes	40 (80.0%)	5 (10.0%)	**< 0.001**
No	10 (20.0%)	45 (90.0%)	
Splenectomy			
Yes	12 (24.0%)	0 (0.0%)	< 0.001
No	38 (76.0%)	50 (100%)	

*β-TM* Beta thalassemia major, *β-TI* Beta thalassemia intermedia, *SD* Standard deviation,

Regarding laboratory features, as illustrated in (Table 3), there was a significant decrease in RBCs, Hb, HCT, and RDW in both β-TM and β-TI groups in comparison with the control group (*P* < 0.001 for all indices). Furthermore, compared to the β-TI group, these indices showed lower significant differences in the β-TM patients (*P* = 0.006 for RDW and *P* < 0.001 for other indices). MCV was significantly decreased in the β-TM group compared to β-TI and control groups (*P* = 0.006 and 0.03, respectively). Both MCH and MCHC showed no significant differences in the studied groups. WBCs significantly increased in the β-TM group compared to the β-TI and controls (*P* < 0.001 for both). Also, WBCs significantly increased in the β-TI group compared to controls (*P* = 0.009). Additionally, there was a significant increase in both serum ferritin and HbF% in the β-TM and the β-TI groups in comparison to the controls (*P* < 0.001). Also, the levels of HbF and ferritin were significantly higher in the β-TM group than in the β-TI group (*P* < 0.001).

**Table 3:** The laboratory features among the studied groups

Laboratory features	β-TM group (No.=50)	β-TI group (No.=50)	Control group (No.=50)	*P* value	Post hoc test
WBCs (x10^3^/cmm) Mean ± SD.	9.2 ± 1.4	4.9 ± 1.3	4.3 ± 0.50	**< 0.001**	**P1 < 0.001**
					**P2 < 0.001**
					**P3 = 0.009**
RBCs (x10^6^/cmm) Mean ± SD	2.8 ± 0.49	3.3 ± 0.79	4.8 ± 0.40	**< 0.001**	**P1 < 0.001**
					**P2 < 0.001**
					**P3 < 0.001**
Hb (g/dl) Mean ± SD	7.2 ± 1.1	8.7 ± 1.4	11.8 ± 0.96	**< 0.001**	**P1 < 0.001**
					**P2 < 0.001**
					**P3 < 0.001**
HCT (%) Mean ± SD	20.6 ± 3.4	25.6 ± 4.8	35.4 ± 2.4	**< 0.001**	**P1 < 0.001**
					**P2 < 0.001**
					**P3 < 0.001**
MCV (fl.) Mean ± SD	71.5 ± 5.1	73.6 ± 4.1	75.1 ± 4.3	**0.01**	**P1 = 0.006**
					**P2 = 0.03**
					P3 = 0.60
MCH (pg) Mean ± SD	25.4 ± 2.1	25.9 ± 1.9	26.4 ± 4.2	0.23	P1 = 0.32
					P2 = 0.09
					P3 = 0.48
MCHC (g/dl) Mean ± SD	34.1 ± 1.8	34.8 ± 0.69	35.2 ± 1.1	0.25	P1 = 0.38
					P2 = 0.09
					P3 = 0.18
RDW (fl.) Mean ± SD	13.7 ± 1.5	14.6 ± 1.4	16.5 ± 1.7	**< 0.001**	**P1 = 0.006**
					**P2 < 0.001**
					**P3 < 0.001**
HbF % Median (IQR)	54.3(40.9–80.2)	25.5(20.4–29.4)	0.30(0.2–0.5)	**< 0.001**	**P1 < 0.001**
					**P2 < 0.001**
					**P3 < 0.001**
Ferritin(ng/ml) Median (IQR)	1824.5(1500–3227)	205.5(168.7–280.7)	42.6(35–57.7.7)	**< 0.001**	**P1 < 0.001**
					**P2 < 0.001**
					**P3 < 0.001**

*β-TM* Thalassemia major, *β-TI* Thalassemia intermedia, *IQR* Interquartile range, *SD* Standard deviation, *WBCs* White blood cells, *RBCs* Red blood cells, *Hb* Hemoglobin, *HCT* Hematocrit, *MCV* Mean corpuscular volume, *MCH* Mean corpuscular hemoglobin, *MCHC* Mean corpuscular hemoglobin concentration, *RDW* Red distribution width, *HbF* Hemoglobin F

P1: β-TM group versus β-TI group

P2: β-TM group versus control group

P3: β-TI group versus control group

### The relative expression profiles of *lncRNA-XIST* and *lncRNA-HBBP1* genes

**Fig. 1:**
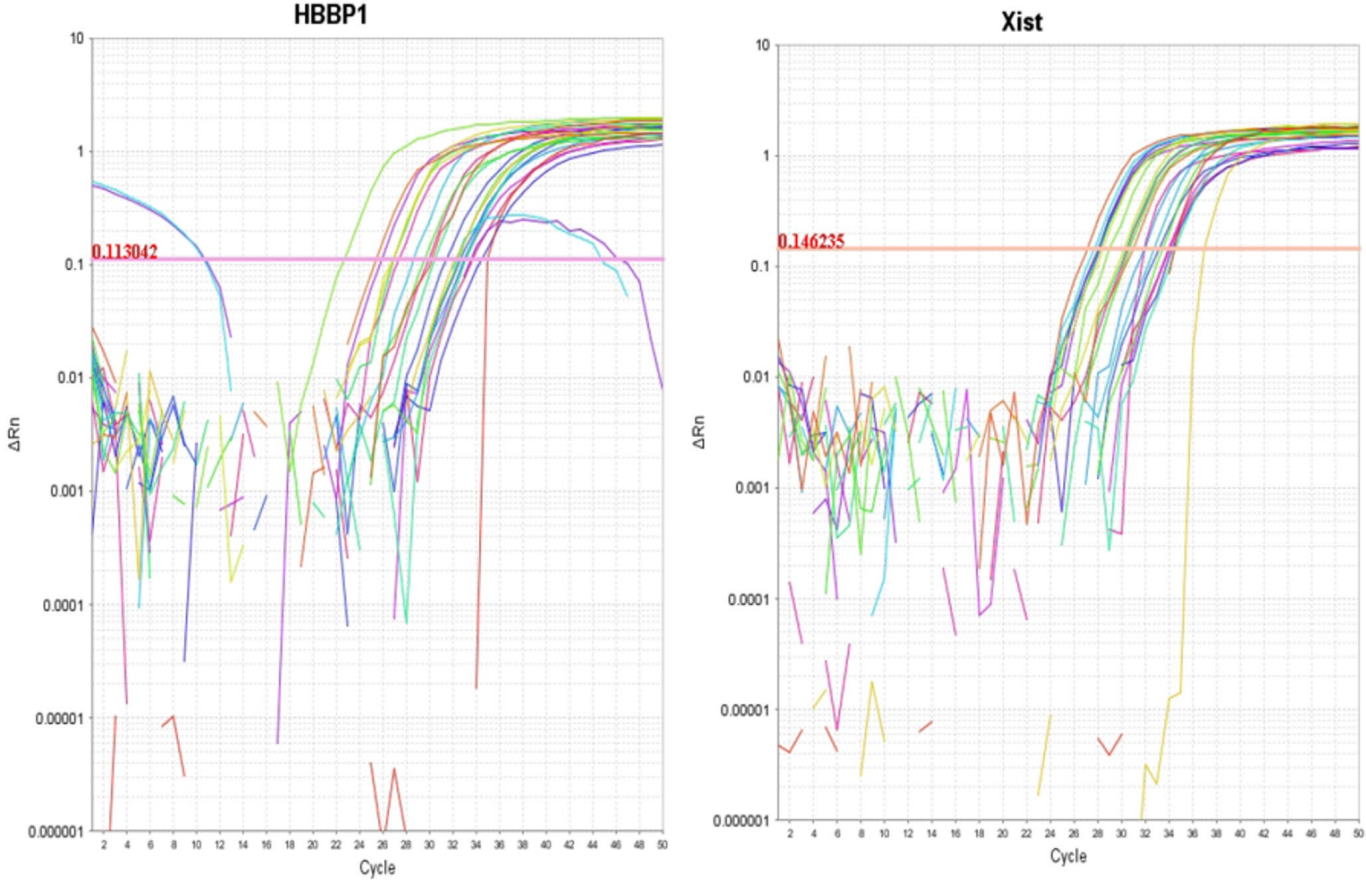
The amplification plots of *lncRNA-HBBP1* and *lncRNA-XIST*

**Fig. 2:**
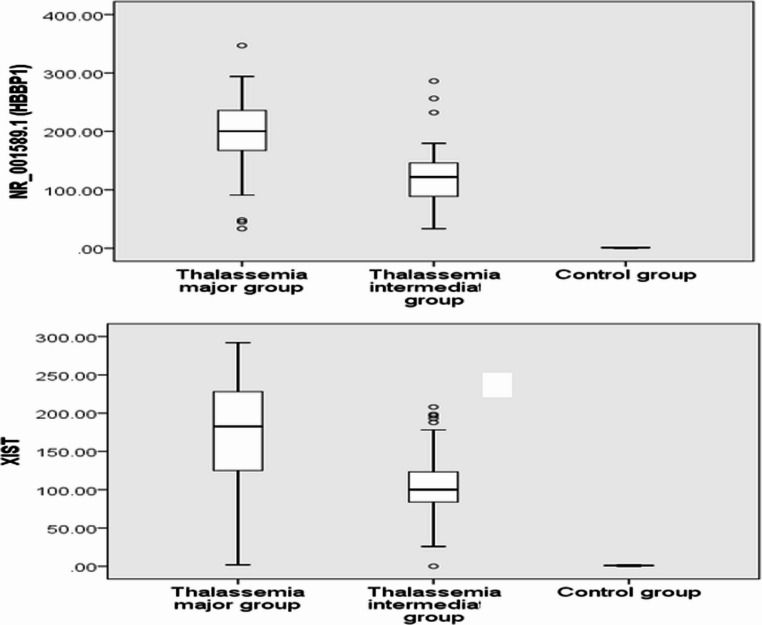
*LncRNA-HBBP1* and *lncRNA-XIST* among the studied groups

RQ (2^−∆∆CT^) was used to assess the expression levels of the genes *lncRNA-XIST* and *lncRNA-HBBP1* among all patients and the controls (Fig. 1). As illustrated in (Table 4; Fig. 2), The RQ values of *lncRNA-HBBP1* and *lncRNA-XIST* were 200.3 (165.6–237.6.6.6) and 182.6 (124.6–229.6.6.6), respectively, in β-TM patients and 122.3 (88.4–146.1) and 100.1 (83.6–124.4), in β-TI patients. The RQ values were 1.04 (0.59–1.4) and 1.1 (0.78–1.2), respectively, in the controls. In the present study, the β-TM group’s expression of *lncRNA-HBBP1* and *lncRNA-XIST* showed a higher significant difference than β-TI and controls (*P* < 0.001 for both). Additionally, both had significantly increased expression in the β-TI group compared to the controls (*p* < 0.001).

**Table 4:** The *lncRNA-HBBP1* and *lncRNA-XIST* expressions among studied groups

	β-TM group (No.=50) Median (IQR)	β-TI group (No.=50) Median (IQR)	Control group (No.=50) Median (IQR)	*P* value	Post hoc test
***lncRNA-HBBP1***	200.3 (165.6–237.6.6.6)	122.03 (88.4–146.1.4.1)	1.1 (0.78–1.2)	*P* < 0.001	**P1 < 0.001** **P2 < 0.001** **P3 < 0.001**
***lncRNA-XIST***	182.6 (124.6–229.6.6.6)	100.1 (83.6- 124.4)	1.04 (0.59–1.4)	*P* < 0.001	**P1 < 0.001** **P2 < 0.001** **P3 < 0.001**

β-TM: Thalassemia major, β-TI: Thalassemia intermedia, *lncRNA*: Long non-coding RNA, *HBBP1*: Hemoglobin subunit β pseudogene 1, *XIST*: X-inactive specific transcript, IQR: Interquartile range,

P1: β-TM group versus β-TI group

P2: β-TM group versus control group

P3: β-TI group versus control group

### ROC curve analysis of *lncRNA-HBBP1* and *lncRNA-XIST*

**Fig. 3:**
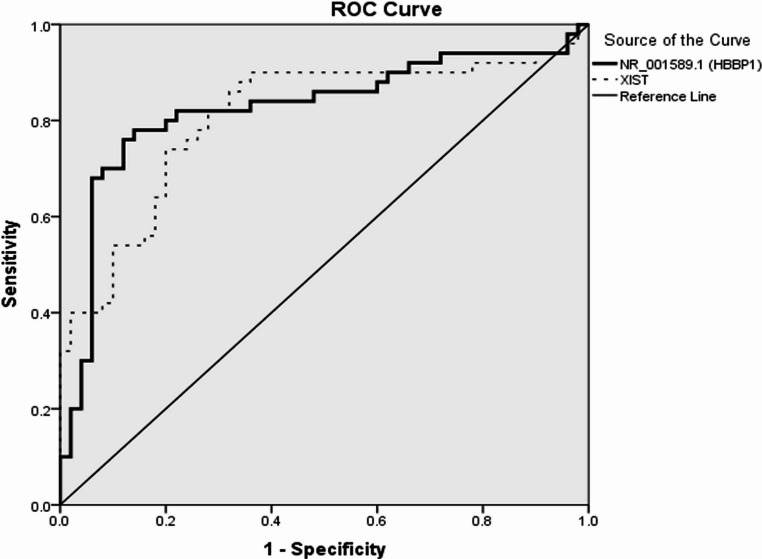
ROC curve of *lncRNA-HBBP1(*NR_001589.1) and *lncRNA-XIST* for β-TM versus β-TI

According to ROC curve analysis for both *lncRNA-HBBP1 and lncRNA-XIST*, the characteristic performance of *lncRNA-HBBP1* showed a cutoff point of 146.67 for β-TM versus β-TI. At this cutoff point, the area under the Curve (AUC) was 0.824, the sensitivity was 82%, and the specificity was 78%. Furthermore, *lncRNA-XIST’*s cutoff point for β-TM compared to β-TI was 122.35, with an AUC of 0.807, sensitivity of 80%, and specificity of 72%, as illustrated in (Table 5; Fig. 3).

**Table 5:** ROC curve of NR_001589.1 (*lncRNA-HBBP1*) and *lncRNA-XIST* for thalassemia major versus thalassemia intermedia

	AUC	Cutoff point	Sensitivity	Specificity	PPV	NPV	Accuracy
***lncRNA-HBBP1*** NR_001589.1	0.824	146.67	82%	78%	78.8%	81.2%	80%
***lncRNA-XIST***	0.807	122.35	80%	72%	74.1%	78.3%	76%

*AUC* Area Under the Curve, *PPV* Positive predictive value, *NPV* Negative predictive value, *lncRNA* Long non-coding RNA, *HBBP1* Hemoglobin subunit β pseudogene 1, *XIST* X-inactive specific transcript

### The impact of *lncRNA-HBBP1* and *lncRNA-XIST* expression on clinical characteristics of β-thalassemia patients

In the current study, as illustrated in (Table 6), the *lncRNA-HBBP1* and *lncRNA-XIST* expression were significantly higher in β-thalassemia patients presented with jaundice, thalassemia facies and organomegaly (*p* < 0.001 for all). In addition, *lncRNA-XIST* expression was significantly higher in patients with splenectomy (*p* = 0.002).

**Table 6:** The impact of *lncRNA-HBBP1* and *lncRNA-XIST* expression on clinical characteristics of β-thalassemia patients

Clinical characteristics	lncRNA-HBBP1 Median (IQR)	*P* value	lncRNA-XIST Median (IQR)	*P* value
Sex		**0.748**		**0.591**
Male	*145.9 (102.6–201.3)*		*125.4 (95.9–189.9)*	
Female	*163.9 (96.8–226.8)*		*123.8 (88.3–189.7)*	
Jaundice		**< 0.001**		**< 0.001**
Yes	267.4 (167.9–334.6)		203.3 (122.9–304.8)	
No	123.5 (87.2–150.04)		96.5 (77.2–121.1)	
Thalassemia facies		**< 0.001**		**< 0.001**
Yes	281.4 (197.2–330.4)		235.8 (159.2–336.9)	
No	128.9 (88.4–186.2)		100.8 (83.6–151.9)	
Organomegaly		**< 0.001**		**< 0.001**
Yes	247 (167.9–301.8)		198.3 (150.4–288.2)	
No	125.7 (88.8–158.6)		100.1 (79.8–123.1)	
Splenectomy		0.18		**0.002**
Yes	250.8 (121.8–300.1)		223.2 (183.8–342.8)	
No	147.5 (97.4–262.9)		105.4 (85.7–199.7)	

Long non-coding RNA, *HBBP1* Hemoglobin subunit β pseudogene 1, *XIST* X-inactive specific transcript

### Prediction of severity of β thalassemia patients

The impact of the studied markers lncRNA-HBBP1 and lncRNA-XIST, and clinical features as jaundice, splenectomy, and organomegaly on TM patients were examined using a logistic regression. The logistic regression model was statistically significant, χ2(4) 110.28, *p* < 0.001. The model explained 89.0% (Nagelkerke R2) of the variance in β thalassemia and correctly classified 50.0% of cases. As illustrated in (Table 7), high expression of lncRNA-HBBP1 and lncRNA-XIST were (4.67 and 8.31 respectively) times more likely to exhibit TM than low expressed patients. Also, Presence of jaundice was linked with TM (OR = 5.18).

**Table 7:** Binary logistic regression analysis for prediction of severity of β Thalassemia patients

Clinical characteristics	*P* value	OR	CI 95%
*lncRNA-HBBP1*	0.002	4.67	3.86–5.64
Jaundice	0.016	5.18	2.07–12.96
*lncRNA-XIST*	0.045	8.31	1.05–68.05

Long non-coding RNA, *HBBP1* Hemoglobin subunit β pseudogene 1, *XIST* X-inactive specific transcript, *OR* Odds ratio, *CI* Confidence Intervals

### The correlation between lncRNA*-HBBP1* and *lncRNA-XIST* gene expression and laboratory features among β-thalassemia patients

A Spearman correlation analysis revealed that both *lncRNA-HBBP1* and *lncRNA-XIST* gene expression were significantly correlated with HbF% (*P* < 0.001 for both) in β- thalassemia patients, either β-TM or β-TI. Additionally, *lncRNA-HBBP1* and *lncRNA-XIST expression* were significantly correlated in both β-TM and β-TI (*P* = 0.02 and < 0.001, respectively). There was no correlation between *lncRNA-HBBP1* and *lncRNA-XIST* gene expression and CBC parameters in both β-TM and β-TI (Table 8).

**Table 8:** Correlation between either *lncRNA-HBBP1* or *lncRNA-XIST* and laboratory parameters among β-TM patients

Laboratory parameters β-TM	LncRNA-HBBP1		LncRNA-XIST	
	*r*	*P* value	*r*	*P* value
WBCs (x10^3^/cmm)	−0.202	0.16	−0.175	0.22
RBCs (x10^6^/cmm)	0.015	0.92	0.031	0.83
HB (g/dl)	−0.010	0.94	−0.170	0.24
HCT %	−0.027	0.85	−0.102	0.48
MCV (fl.)	−0.123	0.39	−0.335	0.1
MCH (pg)	0.014	0.92	−0.157	0.28
MCHC (g/dl)	0.057	0.69	−0.038	0.79
RDW (fl.)	−0.148	0.31	−0.032	0.82
HbF %	0.601	**< 0.001**	0.768	**< 0.001**
Ferritin (ng/ml)	0.015	0.92	−0.208	0.15
Frequency of blood transfusion/year	0.065	0.65	−0.109	0.45
LncRNA-HBBP1	-	-	0.318	**0.02**
Laboratory parameters β-TI	***LncRNA-HBBP1***		***LncRNA-XIST***	
	r	P value	r	P value
WBCs (x10^3^/cmm)	0.14	0.11	0.12	0.1
RBCs (x10^6^/cmm)	−0.040	0.78	0.001	0.99
HB (g/dl)	−0.112	0.44	−0.025	0.86
HCT %	−0.074	0.61	0.027	0.85
MCV (fl.)	−0.156	0.28	0.097	0.50
MCH (pg)	−0.125	0.39	−0.084	0.56
MCHC (g/dl)	0.071	0.63	0.105	0.47
RDW (fl.)	0.263	0.06	0.164	0.26
HbF %	0.654	**< 0.001**	0.560	**< 0.001**
Ferritin (ng/ml)	−0.016	0.91	−0.093	0.52
Frequency of blood transfusion/year	0.083	0.57	0.022	0.88
LncRNA-HBBP1	-	-	0.504	**< 0.001**

*β-TM* Thalassemia major, *β-TI* Thalassemia intermedia, Long non-coding RNA, *HBBP1* Hemoglobin subunit β pseudogene 1, *XIST* X-inactive specific transcript, *WBCs* White blood cells, *RBCs* Red blood cells, *Hb* Hemoglobin, *HCT* Hematocrit, *MCV* Mean corpuscular volume, *MCH* Mean corpuscular hemoglobin, *MCHC* Mean corpuscular hemoglobin concentration, *RDW* Red distribution width, *HbF* Hemoglobin F

## Discussion

Despite advances in understanding the genetic and biochemical etiology of thalassemia, its social and economic effects remain a major concern [23]. High-throughput technology has shown that epigenetic processes, including lncRNAs, greatly affect thalassemia pathogenesis. Identified lncRNAs may lead to novel β-thalassemia diagnostic and treatment strategies [2]. The study’s goals were to establish lncRNA-XIST and lncRNA-HBBP1’s predictive power in thalassemia development and their clinical value in diagnosis.

Both the β-TM and β-TI groups had considerably reduced BMI and weight than the controls in this research. There was no significant difference among studied groups concerning height, sex, and age. According to Sheikh et al., 2017 [23], β-TM patients had a shorter age range (2–16 years) than other groups. Baghiani Moghadam et al., 2011 [24] and Hammod et al., 2018 [25] reported that males were more affected by β-thalassemia than females. Salih and Al-Mosawy, 2013 [26] reported that most patients with β-thalassemia were underweight, supporting our findings. One research found that 60% of thalassemic individuals are underweight and had a substantially lower BMI (*p* < 0.05) than healthy controls across all age groups [27].

The β-TM group in this study showed significantly lower RBCs, Hb, HCT, MCV, and RDW than β-TI and control groups. Similarly, a study by Al-Fatlawi and Al-Safi, 2020 [28] showed a decrease in RBCs count. The cause could be related to the short half-life of RBCs or a malfunction in the bone marrow’s erythropoiesis process, which results from massive destruction of abnormal RBCs within reticular endothelial systems, particularly the spleen organ, precipitating oxidative damage of the cell membrane and interfering with function and survival the red cell maturation [29].

A study by Khawaji et al., 2020 [30] demonstrated a decline in Hb level, which may be related to microcytic anemia with irregular-shaped and decreased RBCs. This leads to hypoxia in the blood and then in the tissues, in which RBC indices decrease (MCHC, MCV, and MCH). This data agreed with what was found by Galanello and Origa [31] and Borgna and Gamberini [32]. Al-fatlawi and AlSafi, 2020 [28] found a low level of HCT observed in β-thalassemia patients in both studied groups because of mutations that occur in the gene responsible for the formation of protein globin, which then leads to RBC becoming fragile and rupturing before maturing [28].

The current study showed a significant rise in WBCs within β-TM patients compared to both β-TI and control groups. WBC counts were significantly increased after splenectomy, and this can be attributed to the stimulation of the hormone erythropoietin (EPO) secretion that causes activated hematopoietic stem cells, which are found in bone marrow, to increase RBCs production, or it may result from a blood transfusion that causes the activity of the immune system [33]. Our results were close to the results of Roth et al., 2018 [34], who found β-TM patients had hypochromic microcytic anemia with a significant decrease in Hb, MCV, MCH, MCHC, and HCT while showing a significant increase in RDW [34].

It was reported that some lncRNAs are implicated in regulating erythropoiesis circuitry, which is tightly linked to β-thalassemia. However, the exact controlling mechanism of these lncRNAs on the transcription of the β-globin gene is not fully understood [35]. In a microarray study, lncRNAs patterns were expressed differentially in β-TM, β-TI and controls. Their results showed that *lncRNA-XIST* had the highest upregulation and showed a significant difference between β-TM patients, and normal controls [6].

In our study, the expression levels of *lncRNA-HBBP1* (NR_001589.1) gene were significantly higher in the β-TM group than the β-TI group and controls. Similarly, Jia et al., 2019 [36] performed RT-qPCR to detect levels of 2 lncRNAs (NR_001589 and uc002fcj.1) expressions in high-HbF patients (β-thalassemia and Hereditary Persistence of Fetal Hemoglobin), they found advanced expression levels of their studied lncRNAs in the high HbF patients in comparison to the control subjects [36]. 

Subsequent investigations emphasized the significance of *lncRNA-HBBP1* during not only physiological but also pathological erythropoiesis [37]. Moreover, several hematopoietic lineage-specific pseudogenes exhibited a robust association with the ancestry-specific master transcription factors; besides, they showed disease correlations, signifying their functional role in human hematopoiesis. Consequently, they highlighted the importance of the functional pseudogenes and the necessities for further developing functional analyses of these pseudogenes [37].

A wide range of clinical symptoms might be found in both homozygotes and compound heterozygotes of the β-thalassemia alleles. These symptoms can range from a severe condition that requires regular blood transfusions, starting in early infancy, to a comparatively milder phenotype. The variability observed is mainly attributed to inconsistency in the globin chains’ biosynthesis, which can be results of β-globin gene mutations or the interaction between the mutant gene and the α or β loci [25]. Mutations in the β-globin gene alone cannot fully account for the phenotypes of β-thalassemia, as often an identical mutation can result in a markedly different phenotype. Epigenetic processes, such as DNA methylation and histone acetylation, could have a role in the development of Mediterranean anemia [24].

We defined the profile of expression levels of *lncRNA-XIST and lncRNA-HBBP1* in β-thalassemia and examined their association with the clinical phenotype. Our data demonstrated that both lncRNAs were positively correlated with high HbF, which could ameliorate the clinical severity of β-thalassemia, and we also found that both genes were positively correlated with each other. These were consistent with previous findings by Lai et al. [14]. Furthermore, Ma et al. [6] discovered that *lncRNA-XIST* is linked to various signaling pathways related to the three systems connected to the pathogenic effects of β-thalassemia: hematologic, skeletal, addition, to hepatic systems via coding-non-coding gene co-expression network and gene ontology process [6].

Our study may be the first to demonstrate a cutoff value for the studied *lncRNA*s that could discriminate between β-TM and β-TI. ROC curve analysis showed that the cutoff point of *lncRNA-HBBP1* for β-TM versus β-TI was 146.67, the sensitivity was 82%, and the specificity was 78%. Also, the cutoff point of *lncRNA-XIST* for β-TM versus β-TI was 122.35, with a sensitivity of 80% and a specificity of 72%. The AUC of *lncRNA-HBBP1* was 0.824, and for *lncRNA-XIST* 0.807. Fakhr-Eldeen et al., 2019 [38] although reporting an overexpression of three other different lncRNAs, namely: myocardial infarction-associated transcript (*MIAT*), metastasis-associated lung adenocarcinoma transcript (*MALAT1*), and antisense noncoding RNA in the INK locus (*ANRIL*) in β-thalassemia patients, they failed to demonstrate a distinguishing level of sensitivity or specificity between β-TM and β-TI [38].

A limitation of this study is the reliance on transfusion dependency and electrophoresis to classify patients into β-TM and β-TI groups. The addition and correlation of genetic mutations and standardized clinical severity with lncRNA expressions may improve disease comprehension in future investigations.

## Conclusions

LncRNA-HBBP1 and lncRNA-XIST may be expressed during β-thalassemia development, providing insight into its molecular genesis. *LncRNA-HBBP1* and *lncRNA-XIST* were overexpressed in β-TM in comparison to β-TI and controls. The expression profiles of these genes could distinguish between β-TM and β-TI with a sensitivity of 82% for *lncRNA-HBBP1* and 80% for *lncRNA-XIST*. Collectively, the examined lncRNAs could offer novel biological markers for β-thalassemia once confirmed in extensive upcoming investigations.

## Recommendations

Additional studies with large sample sizes, including those examining genetic mutations, are generally recommended to confirm the clinical utility of these lncRNAs.

## Data Availability

No datasets were generated or analysed during the current study.

## Abbreviations

β-thalassemia: Beta-thalassemia
lncRNAs: long non-coding RNAs
RNA: Ribonucleic acid
RT-qPCR: Real-time quantitative reverse transcription polymerase chain reaction
HBBP1: Hemoglobin subunit β pseudogene 1
XIST: X-inactive specific transcript
α-thalassemia: alpha thalassemia
NTDT: non-transfusion-dependent thalassemia
CBC: complete blood count
Hb: hemoglobin
BGLT3: Beta Globin Locus Transcript 3
HbD: hemoglobin delta
HNRNPA1: heterogeneous nuclear ribonucleoprotein A1
TAL1: T-cell acute leukemia protein 1
HbF: hemoglobin F
WBCs: white blood cells
RBCs: red blood cells
HCT: hematocrit test, MCV: mean corpuscular volume
BMI: body mass index
WHO: World Health Organization
EDTA: ethylene diamine tetra acetic acid
cDNA: complementary DNA
GAPDH: glyceraldehyde 3-phosphate dehydrogenase
NCBI: National Centre for Biotechnology Information
RQ: Relative quantification
ES: effect size
ROC: Receiver Operator Characteristic Curve
IQR: Interquartile range, SD: Standard deviation
MCH: Mean corpuscular hemoglobin, MCHC: Mean corpuscular hemoglobin concentration, RDW: Red distribution width
AUC: area under the Curve
PPV: Positive predictive value
NPV: Negative predictive value
MIAT: myocardial infarction-associated transcript
MALAT1: metastasis-associated lung adenocarcinoma transcript
ANRIL: antisense noncoding RNA in the INK locus, EPO: erythropoietin, OR: Odds ratio, CI: Confidence Intervals, HbE1:Hemoglobin Subunit Epsilon 1
